# Epithelial tissue folding pattern in confined geometry

**DOI:** 10.1007/s10237-019-01249-8

**Published:** 2019-11-14

**Authors:** Yasuhiro Inoue, Itsuki Tateo, Taiji Adachi

**Affiliations:** 1 Department of Micro Engineering, Kyoto University, Kyoto, Japan; 2 Institute for Frontier Life and Medical Sciences, Kyoto University, Kyoto, Japan

**Keywords:** Epithelial tissue folding, Imaginal primordia development, Multicellular dynamics simulations

## Abstract

The primordium of the exoskeleton of an insect is epithelial tissue with characteristic patterns of folds. As the insect develops from larva to pupa, the spreading of these folds produces the three-dimensional shape of the exoskeleton of the insect. It is known that the three-dimensional exoskeleton shape has already been encoded in characteristic patterns of folds in the primordium; however, a description of how the epithelial tissue forms with the characteristic patterns of folds remains elusive. The present paper suggests a possible mechanism for the formation of the folding pattern. During the primordium development, because of the epithelial tissue is surrounded by other tissues, cell proliferation proceeds within a confined geometry. To elucidate the mechanics of the folding of the epithelial tissue in the confined geometry, we employ a three-dimensional vertex model that expresses tissue deformations based on cell mechanical behaviors and apply the model to examine the effects of cell divisions and the confined geometry on epithelial folding. Our simulation results suggest that the orientation of the axis of cell division is sufficient to cause different folding patterns in silico and that the restraint of out-of-plane deformation due to the confined geometry determines the interspacing of the folds.

**Electronic supplementary material:**

The online version of this article (10.1007/s10237-019-01249-8) contains supplementary material, which is available to authorized users.

## Introduction

Holometabolous insects undergo a remarkable change in surface shape as they grow from larvae to pupae. The mechanism enabling this change is hidden in the structure of imaginal primordia in the larval body. The imaginal primordium comprises epithelial monolayer tissues that are highly folded in a compact manner. Upon the onset of pupation, these folds spread out to form the three-dimensional (3D) shape of the exoskeletal body including appendages of the insect, such as legs and horns.

Folds of epithelial tissue are not limited to the imaginal primordium but are also found in organs of vertebrates, such as brains (Tallinen and Biggins 2015), oviducts (Koyama et al. 2016), and intestines (Hannezo et al. 2011; Krajnc and Ziherl 2015). Tangential expansion of gray matter constrained by white matter is important to the formation of brain folds (Tallinen and Biggins 2015; Tallinen et al. 2016). Intestinal villi are formed by cell proliferation in a monolayer constrained by the underlying stroma. Such a constrained growth of tissue results in compression of the tissue that leads to a mechanical instability such as buckling. Folded shapes are observed in the typical patterns of engineering materials that have buckled (Chan and Crosby 2006; Guvendiren et al. 2010). Buckling is therefore anticipated to be a mechanism of folding epithelial tissues from the mechanical viewpoint. In fact, the tissue folding mentioned above has been explained by mechanical instability arising from constrained tissue growth (Drasdo 2000; Hannezo et al. 2011; Krajnc and Ziherl 2015; Tallinen and Biggins 2015; Tallinen et al. 2016).

Although there is potentially a mechanism of folding epithelial tissues that is common to insects and vertebrate organs, the main difference between the primordium and vertebrate organs is the function of the folded structure. The epithelial tissue of the primordium is premised to unfold to establish 3D exoskeletal shapes at a subsequent stage while vertebrate organs are designed to remain folded for their functionality. Therefore, the folded structure of the imaginal primordium is not the final form of development. There is thus one more mechanism hidden in the structure of imaginal primordia in the larval body that establishes the final 3D shape from the primordium.

To reveal the mechanism of encoding the final 3D shape in the primordium, the relationship between the 3D shape of the pupa and primordia has been clarified in experiments in which the beetle horn primordium of *Trypoxylus dichotomus* has been extracted and observed to spread out using several methods (Matsuda et al. 2017). Those experimental results show that there are characteristic folding patterns, such as labyrinth, stripe, and concentric patterns, and revealed what part of the 3D surface shape of the horn is encoded in these characteristic patterns. In addition, because of the imaginal primordia of the *Drosophila* leg (Morata 2001) and the tip of the beetle horn (Matsuda et al. 2017) have similar (concentric) patterns, there might be a robustness of the relationship between folding patterns and their 3D shapes. However, it is not well understood how these folding patterns of the primordia form in the larval body.

In terms of the development of primordia, because of imaginal primordia are surrounded by other tissues, such as the peripodial membrane (Beira and Paro 2016; Milner et al. 1984), cell proliferation proceeds within a confined geometry. The tissue deformation driven by cell proliferation is thus restrained by the surrounding tissue. Observations of the imaginal primordium of the wings of *Drosophila* have revealed that cells proliferate with a certain orientation of cell division (Mao et al. 2011, 2013). The regulation of the orientation of cell division is the planar cell polarity signaling pathway. In *Drosophila*, planar cell polarity molecules such as dachsous molecules function as global direction cues. In response to the dachsous gradient, Dachs, an atypical myosin, is localized in a planar-polarized manner. Dachs controls the long axis of the cell shape on the apical side and thus regulates the orientation of cell division (Mao et al. 2011). Mutation of the *dachsous* gene results in a wing of *Drosophila* that is shorter and wider than that of the wild type (Baena-López et al. 2005). However, it has been confirmed that randomization of the axis of cell division does not appreciably affect the adult form of *Drosophila* (Zhou et al. 2019). In growing beetle horn primordia, the global shape of the horn primordia is mushroom like, with dense local furrows. The global shape is affected in the *dachsous*-gene knocked-down beetle, in which the direction of cell division has been altered randomly, while dense local furrows are not appreciably affected (Adachi et al. 2018). The exact role of the orientation of cell divisions is therefore not clear in vivo.

During the last decade, mathematical models for the computational simulation of epithelial tissue mechanics have been well established (Hannezo et al. 2011; Heisenberg and Bellaïche 2013; Honda et al. 2004, 2008; Okuda et al. 2013a, b; Takeda et al. 2019) and applied to test hypotheses motivated by biological experiments (Amar and Jia 2013; Eiraku et al. 2011; Fletcher et al. 2014; Inoue et al. 2016, 2017; Okamoto et al. 2013). To suggest a possible role of cell proliferation with a certain orientation of cell division in the confined geometry, we examine whether the above mechanical effects are sufficient to form a proper pattern of folds in silico. We here employ a 3D vertex model (Okuda et al. 2013a) that considers the behavior of an individual cell to express epithelial tissue deformations driven by the activity of each cell. Using the model, we perform computer simulations for several orientations of cell division and magnitudes of the restraint of tissue deformation to examine effects of the orientation of cell division and confined geometry on the formation of folding patterns.

## Three-dimensional vertex model expressing multicellular dynamics

**Fig. 1:**
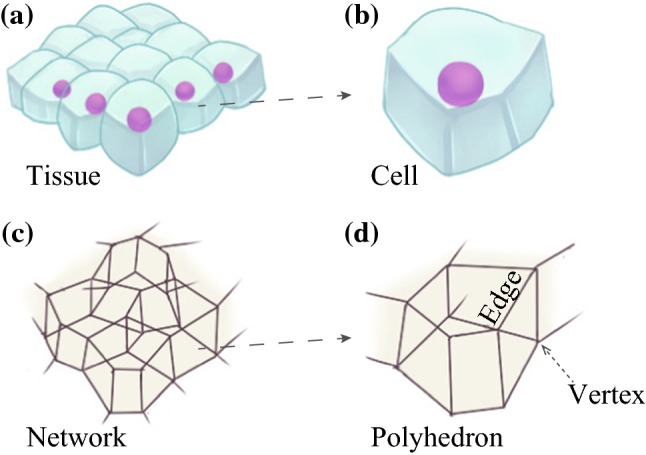
Shapes of **a** epithelial tissue and **b** cells respectively modeled as **c** a network and **d** polyhedrons using the 3D vertex model

The 3D vertex model expresses the shape of a cell as a polyhedron comprising vertices and edges. The tissue is represented as an aggregate of multiple cells (Fig. 1a, b), where the vertices and edges of each cell are shared with neighboring cells. The vertices and edges thus constitute a network that represents the entire shape of the tissue (Fig. 1c, d).

The equation of motion of vertex *i* with position vector ${\mathbf {r}}_i$ at time *t* is

1 $$\begin{aligned} \eta \left( \frac{\mathrm{d}{\mathbf {r}}_i}{\mathrm{d}t}-{\mathbf {V}}_i\right)= & {} -\nabla _i U, \end{aligned}$$

where $\eta$ is the friction coefficient and ${\mathbf {V}}_i$ is the mean velocity vector of vertex *i*. To satisfy the Galilean invariance of the equation of motion, the friction force expressed in Eq. (1) is calculated on a local inertial frame. In the 3D vertex model, vertex *i* is directly connected to four adjacent vertices by edges. Indexing the directly connected vertices as *j*(*i*), the mean velocity vector is defined as

2 $$\begin{aligned} {\mathbf {V}}_i= & {} \frac{1}{5}\left( \frac{\mathrm{d}{\mathbf {r}}_{i}}{\mathrm{d}t}+\sum _{j(i)}\frac{\mathrm{d}{\mathbf {r}}_{j(i)}}{\mathrm{d}t}\right) . \end{aligned}$$

Assuming that all vertices have the same friction coefficient, Eqs. (1) and (2) are derived from the previous 3D vertex model (Okuda et al. 2015).

The right-hand side of Eq. (1) represents a force acting on vertex *i* derived from the total energy function *U*, which represents the mechanical properties and morphogenetic events of the cells. *U* is expressed as

3 $$\begin{aligned} U= & {} \sum _{j^c}^{\mathrm{cell}}\left( u_{j^c}^{\mathrm{v}} + u_{j^c}^{\mathrm{s}} + u_{j^c}^{\mathrm{h}} +u_{j^c}^{\mathrm{r}}\right) + u^{\mathrm{sc}}, \end{aligned}$$

where $\sum _{j^c}^{\mathrm{cell}}$ indicates a summation over all cells. The energy function *U* includes the cell volume elastic energy $u_{j^c}^{\mathrm{v}}$, cell surface elastic energy $u_{j^c}^{\mathrm{s}}$, cell height elastic energy $u_{j^c}^{\mathrm{h}}$, restraint energy with respect to out-of-plane deformation $u_{j^c}^{\mathrm{r}}$, and self-collision energy $u^{\mathrm{sc}}$. Mathematical expressions for these energy functions are

4 $$\begin{aligned} u_{j^c}^{\mathrm{v}}\left( v_{j^c}\right)= & {} \frac{1}{2}k^{\mathrm{v}}\left( \frac{v_{j^c}}{v_{j^c}^{\mathrm{eq}}}-1\right) ^2, \end{aligned}$$

5 $$\begin{aligned} u_{j^c}^{\mathrm{s}}\left( s_{j^c}\right)= & {} \frac{1}{2}k^{\mathrm{s}}\left( \frac{s_{j^c}}{s_{j^c}^{\mathrm{eq}}}-1\right) ^2,\end{aligned}$$

6 $$\begin{aligned} u_{j^c}^{\mathrm{h}}\left( h_{j^c}\right)= & {} \frac{1}{2}k^{\mathrm{h}}\left( \frac{h_{j^c}}{h_{j^c}^{\mathrm{eq}}}-1\right) ^2,\end{aligned}$$

7 $$\begin{aligned} u_{j^c}^{\mathrm{r}}\left( h_{j^c}\right)= & {} \frac{1}{2}k^{\mathrm{r}}s^{\mathrm{a}}_{j^c}z_{j^c}^2,\end{aligned}$$

8 $$\begin{aligned} u^{\mathrm{sc}}= & {} \left\{ \begin{array}{ll} \mathop \sum \nolimits _i^{\mathrm{vertex}}\mathop \sum \nolimits _n^{\mathrm{vertex}}\frac{1}{2}k^{\mathrm{sc}}\left( \frac{l_{in}}{\sigma }-1\right) ^2 &{} (l_{in}<\sigma ) \\ 0 &{} (l_{in}\ge \sigma ) \end{array} \right. . \end{aligned}$$

Equations (4–6) are derived from a 3D vertex model of epithelial cells (Inoue et al. 2016, 2017). The volume $v_{j^c}$, surface area $s_{j^c}$, and height $h_{j^c}$ of the $j^c$th cell are represented as variables. The superscript eq used for several variables in Eqs. (4–6) refers to the value in the stress-free state. The constants $k^{\mathrm{v}}$, $k^{\mathrm{s}}$, and $k^{\mathrm{h}}$ are, respectively, the volume elasticity, surface elasticity, and height elasticity. The cell height is defined as the distance between the centroid of the apical face and that of the basal face of the cell.

The restraint of out-of-plane deformation is introduced in terms of the harmonic potential $u_{j^c}^{\mathrm{r}}$ that is responsible for the restoring force acting on the $j^c$th cell with respect to its out-of-plane displacement, $z_{j^c}$, along the *z*-axis. The mathematical form of $u_{j^c}^{\mathrm{r}}$ is the same as that introduced in (Brau et al. 2013), in which a small out-of-plane displacement of a thin sheet is considered. We assume that growing tissue is in contact with the surrounding tissue on the apical side. We thus use the out-of-plane displacement of the centroid of the apical face of the $j^c$th cell, $z_{j^c}^{\mathrm{a}}$, as $z_{j^c}$. Because the restraint energy is defined for the unit area (Brau et al. 2013), the total restraint energy for the cell is obtained by multiplying by the apical surface area. This is why the restraint coefficient is expressed as the product of the restraint constant, $k^{\mathrm{r}}$, and the surface area of the apical face of the $j^c$th cell, $s^{\mathrm{a}}_{j^c}$. The self-collision energy $u^{\mathrm{sc}}$ is introduced to prevent simulated tissue from penetrating in the direction of collision using a penalty-based method (Tang et al. 2012), where $k^{\mathrm{sc}}$, $l_{in}$, and $\sigma$ are the self-collision energy constant, distance between vertices *i* and *n*, and threshold distance of the collision, respectively.

**Fig. 2:**
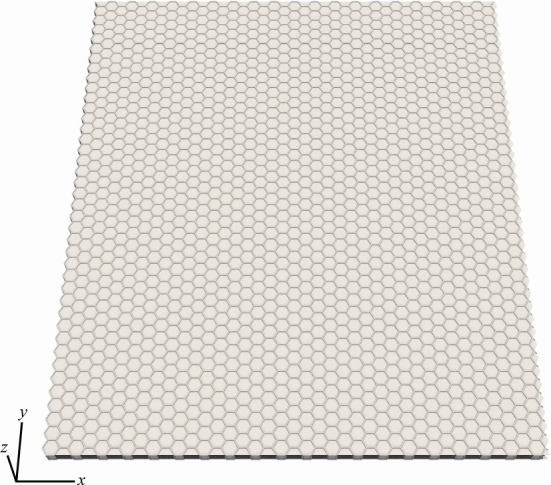
Initial shape of the monolayer cell sheet. The *x*-axis and *y*-axis are defined on the plane of the initial sheet, and the *z*-axis is defined normal to the plane. Periodic boundary conditions are applied for *x* and *y* directions. Cells on the boundaries are not visualized

All model constants are listed in Table 1. Here, to focus on folded structures created by cell proliferation, we ignore the asymmetry of physical properties dependent on epithelial polarity and assume a flat, homogeneous, epithelial monolayer sheet (Fig. 2) for the initial condition. The size of the sheet is $37.22 \times 42.98$ corresponding to $40\times 40$ cells arranged in a regular hexagonal lattice in the $x-y$ plane under initial conditions. Although we have not performed a pre-simulation for equilibration of the system, the initial conditions have been confirmed to be stable in a planar and hexagonal packing of the columnar cells (animation shown in Supplementary Material 1). The apical surface of the sheet is set to face to the +z direction. Periodic boundary conditions are adopted for *x* and *y* directions. To express cell proliferation, we employ a polyhedron-division model (Okuda et al. 2013b), where the timing of cell division is determined by a mean cell cycle time $\tau _{\mathrm{cc}}$ and standard deviation $\sigma _{\mathrm{cc}}$.

**Table 1:** Model constants

Symbol	Value	Descriptions
$k^{\mathrm{v}}$	20	Volume elasticity
$k^{\mathrm{s}}$	0.256	Area elasticity
$k^{\mathrm{h}}$	0.1	Height elasticity
$k^{\mathrm{r}}$	0.0–$1.6\times 10^{-2}$	Restraint constant
$k^{\mathrm{sc}}$	40	Self-collision energy constant
$v^{\mathrm{eq}}$	1.0	Cell volume at stress free state
$s^{\mathrm{eq}}$	$2v^{\mathrm{eq}}/h^{\mathrm{eq}}+2\sqrt{2\sqrt{3}v^{\mathrm{eq}}h^{\mathrm{eq}}}$	Cell surface area (hexagonal prism) at stress free state
$h^{\mathrm{eq}}$	1.0	Cell height at stress free state
$\sigma$	1.0	Collision threshold distance
$\tau _{\mathrm{cc}}$	1000	Mean cell cycle time
$\sigma _{\mathrm{cc}}$	10	Standard deviation of the cell cycle time
$\eta$	0.25	Friction coefficient of vertex
$\varDelta t$	$2.0\times 10^{-4}$	Time step size for numerical integration of Eq. (1)

## Results

### Folding pattern obtained for the orientation of cell division in a confined geometry

**Fig. 3:**
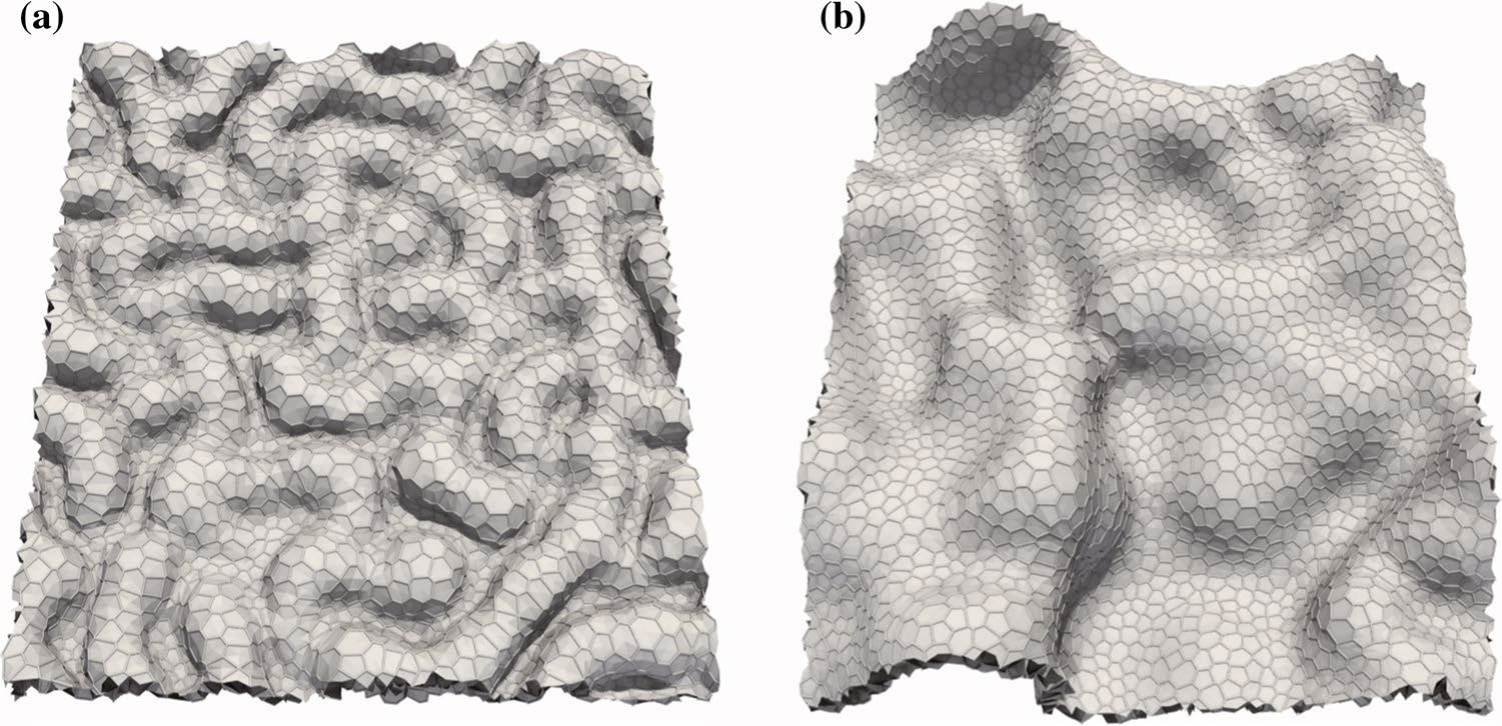
Snapshots of epithelial folding simulated under the conditions of **a** restraint of out-of-plane deformation ($k^{\mathrm{r}}= 1 \times 10^{-3}$) and **b** no restraint of out-of-plane deformation ($k^{\mathrm{r}} = 0$). All snapshots show simulation results at time $t = 0.5 \tau _{\mathrm{cc}}$

We perform 3D vertex simulations of epithelial tissue growth to examine whether tissue growth in a confined geometry produces a folded tissue structure. Simulation results in Fig. 3 show that the restraint of out-of-plane deformation results in a short interspacing of the folds (Fig. 3a, animation shown in Supplementary Material 2) as compared with the interspacing under the condition of no restraint (Fig. 3b, Suppl. Mov. 3).

**Fig. 4:**
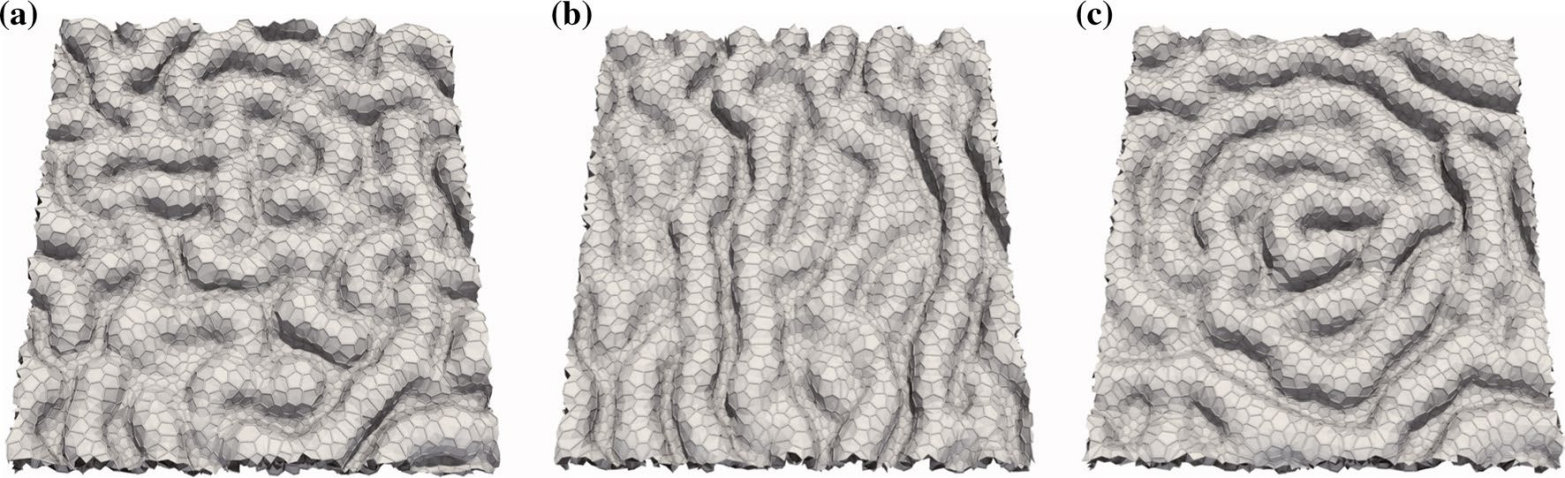
Snapshots of epithelial folding simulated for three orientations of the cell division axis: **a** longest axis of each cell, **b** uniaxial direction (*x*-axis), and **c** radial axis ($k^{\mathrm{r}} = 1.0\times 10^{-3}$). All snapshots show simulation results at time $t = 0.5 \tau _{\mathrm{cc}}$

We next investigate how the orientation of the cell division axis affects the folded structure. We consider three orientations of the cell division axis: the longitudinal direction of each cell shape, uniaxial direction in global coordinates, and radial direction toward the centroid of the tissue. The simulation results in Fig. 4 show that (a) random patterns of folds form for the longitudinal orientation (animation shown in Supplementary Material 2), (b) folds align in one direction like stripes for the uniaxial orientation (Suppl. Mov. 4), and (c) folds form a concentric pattern for the radial orientation of cell division (Suppl. Mov. 5). The interspacing of folds is almost the same regardless of the orientation of the axis of cell division (with detailed analysis presented in the next section), suggesting that the orientation of the cell division axis plays a role in determining the pattern of folds.

### Length scale of folds determined by the restraint of out-of-plane deformation

**Fig. 5:**
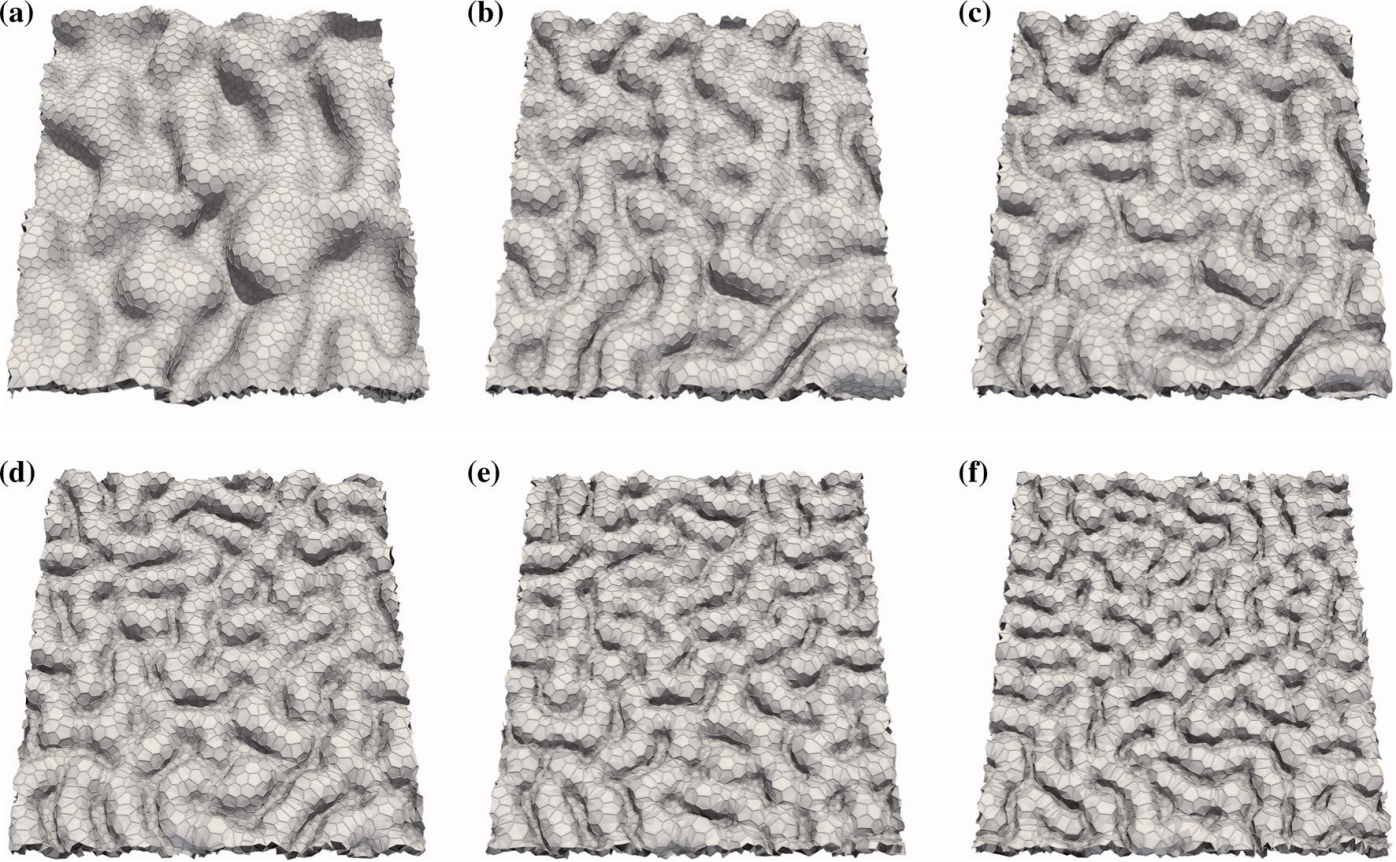
Snapshots of epithelial folding simulated under the conditions of **a**$k^{\mathrm{r}} = 6.25\times 10^{-5}$ , **b**$k^{\mathrm{r}} = 5\times 10^{-4}$, **c**$k^{\mathrm{r}} = 1\times 10^{-3}$, **d**$k^{\mathrm{r}} = 2\times 10^{-3}$, **e**$k^{\mathrm{r}} = 4\times 10^{-3}$, and **f**$k^{\mathrm{r}} = 8\times 10^{-3}$. The orientation of cell division is along the longest axis of each cell. All snapshots show simulation results at time $t=0.5 \tau _{\mathrm{cc}}$

We examine the effect of the restraint constant, $k^{\mathrm{r}}$, on the interspacing of folds to determine how the length scale of the folds is affected. The simulation results in Fig. 5 show that an increase in $k^{\mathrm{r}}$ leads to shorter interspacing of the folds. To quantify the interspacing, we analyze the wavenumber, *u*, of the fold pattern using the Fourier transformation and obtain the mean wavenumber, ${\overline{u}}$, as a weighted average of *u* using the Fourier coefficient.

**Fig. 6:**
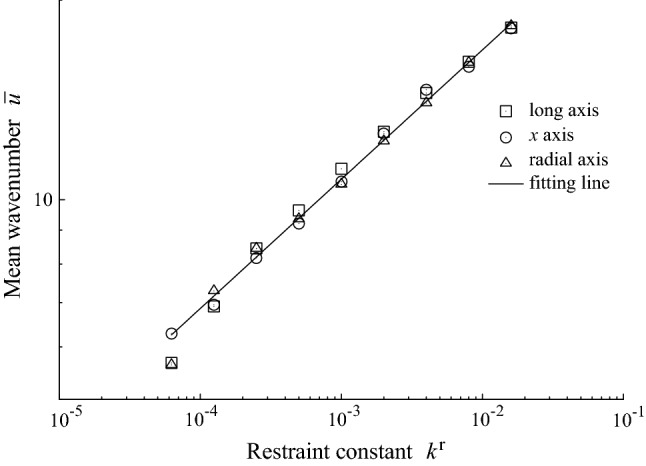
Relationship between the mean wavenumber and restraint constant. The legend gives the orientation of cell division. The line shows the least-squares fitting line: $\log {\bar{u}} = 0.201\log k^{\mathrm{r}}+3.76$

Figure 6 shows the power law relationship between the mean wavenumber and restraint constant. All data points obey a unique power law regardless of the orientation of the axis of cell division, suggesting that interspacing of the folds can be determined by the restraint of the out-of-plane deformation due to the surrounds of the growing tissue.

The characteristic wavenumber of folds has been derived theoretically whereby the balance of normal forces under uniaxially compressed sheet registing on a soft substrate is considered (Brau et al. 2013). In the theory (Cerda and Mahadevan 2003; Brau et al. 2013), assuming that the bending stiffness of the tissue and the restraint constant are independent of the size of deformation, the power exponent could be 0.25, while the power exponent is approximately 0.20 in our simulations. Because of the restraint constant in our simulation is independent of the size of deformation, we speculate that a discrepancy between the exponent in simulation and that in theoretical prediction is due to discreetness originating from the cell size. Because of the tissue bending will not be realized on a length scale shorter than the cell width, the wavenumber is upper bounded by the corresponding wavenumber of the cell width. In fact, applying least-squares fitting to data in the small wavenumber range (${\bar{u}} \le 10$), we obtained the power exponent of 0.25 (Appendix).

In addition to the discreetness originating from the cell size, because of the bending stiffness of the tissue is not given directly in the simulation but induced by the combination of the energy functions of the cell, there is the possibility that the bending stiffness effectively depends on the wavenumber of the deformation. To explain the power exponent of 0.20 in this simulation using the theory (Brau et al. 2013), it is speculated that the bending stiffness is proportional to the wavenumber if we consider the independence of the restraint constant on the wavenumber. It is future work to use a 3D vertex model to derive the mechanical properties of the tissue from cell energy functions.

## Discussion

We showed that the orientation of cell division and the restraint of out-of-plane deformation are respectively sufficient to cause the characteristic pattern and interspacing of folds in silico. The three patterns of folds obtained in this study coincide with the characteristic pattern of folds observed in beetle horns (Matsuda et al. 2017). The concentric pattern of folds is also found in the imaginal disk of *Drosophila* legs (Beira and Paro 2016; Schubiger et al. 2012).

However, we do not insist that the mechanism proposed in this study is the only mechanism determining the folding pattern. The actual beetle horn is cylindrical and there is thus a possibility that there is a mechanical difference in the ease of forming folds in the direction of the cylinder axis and in the circumferential direction. In fact, curvature-inducing wrinkles have been pointed out in stiff thin film on curved soft substrates (Stoop et al. 2015), and a similar mechanism might be available for actual beetles.

Although the global shape of the beetle horn primordia is affected in the *dachsous*-gene knocked-down beetle, in which the direction of cell division has been altered randomly, dense local furrows are not appreciably affected (Adachi et al. 2018). In the development of beetle primordia, actin accumulation, indicating apical constrictions of cells, has been observed at the position of a future furrow even before the local furrow forms, suggesting pre-patterning for the fine furrow pattern (Adachi et al. 2018).

Furthermore, it has been confirmed that perturbation of the axis of cell division does not appreciably affect the adult form of *Drosophila* (Zhou et al. 2019). Although a spatial pattern of actomyosin accumulation at a supracellular scale has not been reported for *Drosophila* primordia development, the spatial pattern of actomyosin accumulation or a still unknown mechanism may be considered for the primordia development of such small-sized bodies and tips of appendages. We speculate that there are at least two mechanisms that determine global and local folding patterns. The relationship between the spatial pattern of apical constriction and the 3D shape of deformed tissues has been investigated using a 3D vertex model (Inoue et al. 2017) and there are thus future opportunities to combine the present model with the pattern of apical constriction and to apply the model to clarify how these two mechanisms do or do not crosstalk.

In this study, the effect of confinement was introduced by the restraint of out-of-plane deformation on the apical side solely using Eq. (7). In addition to the apical side, it is possible to introduce the effect of confinement on the basal side, such as in the case of an extracellular matrix, using Eq. (7) and replacing the apical surface area term with a basal surface area term. However, the simulation results obtained when restraining both apical and basal sides coincide with those obtained when restraining the apical side solely in terms of there being an effective restraint constant that is the sum of values for the restraint constants on apical and basal sides (Appendix). With respect to introducing the effect of confinement, another possible model is tissue bounded by rigid walls on apical and basal sides. Because the rigid wall cannot be displaced or deformed, the interspace between the tissue and wall under initial conditions is an important parameter with which to determine tissue deformation. There is future opportunity to clarify how the interspace determines the tissue folding.

### Electronic supplementary material

Below is the link to the electronic supplementary material.

Supplementary material 1 (pdf 151 KB)

Supplementary material 2 (mp4 4100 KB)

Supplementary material 3 (mp4 7638 KB)

Supplementary material 4 (mp4 7644 KB)

Supplementary material 5 (mp4 7747 KB)

Supplementary material 6 (mp4 7679 KB)

## Figures and Tables

**Fig. 7:**
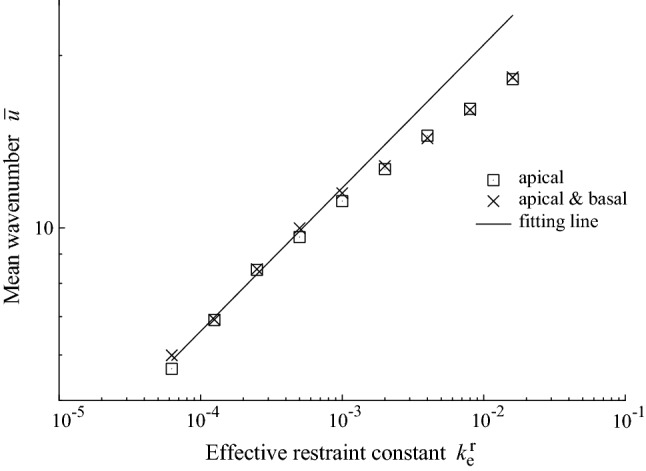
Relationship between the mean wavenumber and restraint constant. The legend gives the confinement side. The effective restraint constant, $k^{\mathrm{r}}_{\mathrm{e}}$, is the sum of the apical and basal restraint constants. The line shows the least-squares fitting line for small wavenumbers (${\bar{u}} \le 10$): $\log {\bar{u}} = 0.252\log k^{\mathrm{r}}+4.20$. Simulation results are obtained for the longest axis of cell division

## Appendix: Confined tissue growth on apical and basal sides

In this study, we assumed that growing tissue is confined by the surrounding tissue on the apical side. We therefore examined whether tissue folding is different if the growing tissue is confined on apical and basal sides. Instead of Eq. (7), we applied the energy function

9 $$\begin{aligned} u_{j^c}^{\mathrm{r}}\left( h_{j^c}\right)= & {} \frac{1}{2}k^{\mathrm{r}}s^{\mathrm{a}}_{j^c}{z_{j^c}^{\mathrm{a}}}^2 + \frac{1}{2}k^{\mathrm{r}}s^{\mathrm{b}}_{j^c}{z_{j^c}^{\mathrm{b}}}^2 \text{, } \end{aligned}$$

where we introduce the out-of-plane displacement of the basal face and basal surface area of the $j^c$th cell as $z_{j^c}^{\mathrm{b}}$ and $s^{\mathrm{b}}$, respectively.

Figure 7 shows the relationship between the mean wavenumber of interspacing of folds and an effective restraint constant, $k_{\mathrm{e}}^{\mathrm{r}}$, where $k_{\mathrm{e}}^{\mathrm{r}}$ is $k^{\mathrm{r}}$ and $2k^{\mathrm{r}}$ for tissue confined on the apical side solely and tissue confined on both apical and basal sides, respectively. The results obtained using the apical and basal constraints coincide with those obtained using the apical constraint solely in terms of $k_{\mathrm{e}}^{\mathrm{r}}$. We explain this result in the following. The cell height is constant because of the cell height elastic energy given by Eq. (6). Therefore, the out-of-plane displacement of apical and basal centroids is the same as that of the cell centroid $z_{j^c} = z_{j^c}^{\mathrm{a}} = z_{j^c}^{\mathrm{b}}$. In the case of small out-of-plane deformation, we assume that the apical surface area is the same as that of the basal surface area $s^{\mathrm{a}} = s^{\mathrm{b}}$. Equation (9) thus reduces to

10 $$\begin{aligned} u_{j^c}^{\mathrm{r}}\left( h_{j^c}\right)= & {} \frac{1}{2}k_{\mathrm{e}}^{\mathrm{r}}s^{\mathrm{a}}_{j^c}z_{j^c}^2 \text{, } \end{aligned}$$

where $k^{\mathrm{r}}_{\mathrm{e}} = 2k^{\mathrm{r}}$ for tissue confined on apical and basal sides. Simulations with the same value of $k_{\mathrm{e}}^{\mathrm{r}}$ provide the same result regardless of the confinement side.

In addition, by applying least-squares fitting in the case of small wavenumbers (${\bar{u}} \le 10$), we obtain a power exponent of 0.252. We therefore consider that the discrepancy in the power exponent between our simulation results and the theoretical prediction arises mainly from discreteness originating from the cell size.

## References

[CR1] Adachi H, Matsuda K, Niimi T (2018). Anisotropy of cell division and epithelial sheet bending via apical constriction shape the complex folding pattern of beetle horn primordia. Mech Dev.

[CR2] Amar MB, Jia F (2013). Anisotropic growth shapes intestinal tissues during embryogenesis. PNAS.

[CR3] Brau F, Damman P, Diamant H, Witten TA (2013). Wrinkle to fold transition: influence of the substrate response. Soft Matter.

[CR4] Baena-López LA, Baonza A, García-Bellido A (2005). The orientation of cell divisions determines the shape of Drosophila organs. Curr Biol.

[CR5] Beira JV, Paro R (2016). The legacy of *Drosophila* imaginal discs. Chromosoma.

[CR6] Cerda E, Mahadevan L (2003). Geometry and physics of wrinkling. Phys Rev Lett.

[CR7] Chan EP, Crosby AJ (2006). Fabricating microlens arrays by surface wrinkling. Adv Mater.

[CR8] Drasdo D (2000). Buckling instabilities of one-layered growing tissues. Phys Rev Lett.

[CR9] Eiraku M, Takata N, Ishibashi H (2011). Self-organizing optic-cup morphogenesis in three-dimensional culture. Nature.

[CR10] Fletcher AG, Osterfield M, Baker RE, Shvartsman SY (2014). Vertex models of epithelial morphogenesis. Biophys J.

[CR11] Guvendiren M, Burdick JA, Yang S (2010). Kinetic study of swelling-induced surface pattern formation and ordering in hydrogel films with depth-wise crosslinking gradient. Soft Matter.

[CR12] Hannezo E, Prost J, Joanny J-F (2011). Instabilities of monolayered epithelia: shape and structure of villi and crypts. Phys Rev Lett.

[CR13] Heisenberg C-P, Bellaïche Y (2013). Forces in tissue morphogenesis and patterning. Cell.

[CR14] Honda H, Tanemura M, Nagai T (2004). A three-dimensional vertex dynamics cell model of space-filling polyhedra simulating cell behavior in a cell aggregate. J Theor Biol.

[CR15] Honda H, Motosugi N, Nagai T (2008). Computer simulation of emerging asymmetry in the mouse blastocyst. Development.

[CR16] Inoue Y, Suzuki M, Watanabe T (2016). Mechanical roles of apical constriction, cell elongation, and cell migration during neural tube formation in Xenopus. Biomech Model Mechanobiol.

[CR17] Inoue Y, Watanabe T, Okuda S, Adachi T (2017). Mechanical role of the spatial patterns of contractile cells in invagination of growing epithelial tissue. Dev Growth Differ.

[CR18] Koyama H, Shi D, Suzuki M (2016). Mechanical regulation of three-dimensional epithelial fold pattern formation in the mouse oviduct. Biophys J.

[CR19] Krajnc M, Ziherl P (2015). Theory of epithelial elasticity. Phys Rev E.

[CR20] Mao Y, Tournier AL, Bates PA (2011). Planar polarization of the atypical myosin Dachs orients cell divisions in *Drosophila*. Genes Dev.

[CR21] Mao Y, Tournier AL, Hoppe A (2013). Differential proliferation rates generate patterns of mechanical tension that orient tissue growth. EMBO J.

[CR22] Matsuda K, Gotoh H, Tajika Y (2017). Complex furrows in a 2D epithelial sheet code the 3D structure of a beetle horn. Sci Rep.

[CR23] Morata G (2001). How drosophila appendages develop. Nat Rev Mol Cell Biol.

[CR24] Milner MJ, Bleasby AJ, Kelly SL (1984). The role of the peripodial membrane of leg and wing imaginal discs of *Drosophila* melanogaster during evagination and differentiation in vitro. Wilhelm Roux’ Archiv.

[CR25] Okamoto M, Namba T, Shinoda T (2013). TAG-1-assisted progenitor elongation streamlines nuclear migration to optimize subapical crowding. Nat Neurosci.

[CR26] Okuda S, Inoue Y, Eiraku M (2013). Reversible network reconnection model for simulating large deformation in dynamic tissue morphogenesis. Biomech Model Mechanobiol.

[CR27] Okuda S, Inoue Y, Eiraku M (2013). Modeling cell proliferation for simulating three-dimensional tissue morphogenesis based on a reversible network reconnection framework. Biomech Model Mechanobiol.

[CR28] Okuda S, Inoue Y, Eiraku M (2015). Vertex dynamics simulations of viscosity-dependent deformation during tissue morphogenesis. Biomech Model Mechanobiol.

[CR29] Schubiger G, Schubiger M, Sustar A (2012). The three leg imaginal discs of *Drosophila*: “Vive la différence”. Dev Biol.

[CR30] Stoop N, Lagrange R, Terwagne D (2015). Curvature-induced symmetry breaking determines elastic surface patterns. Nat Mater.

[CR31] Takeda H, Kameo Y, Inoue Y, Adachi T (2019). An energy landscape approach to understanding variety and robustness in tissue morphogenesis. Biomech Model Mechanobiol.

[CR32] Tallinen T, Biggins JS (2015). Mechanics of invagination and folding: Hybridized instabilities when one soft tissue grows on another. Phys Rev E Stat Nonlinear Soft Matter Phys.

[CR33] Tallinen T, Chung JY, Rousseau F (2016). On the growth and form of cortical convolutions. Nat Phys.

[CR34] Tang M, Manocha D, Otaduy MA, Tong R (2012). Continuous penalty forces. ACM Trans Graph.

[CR35] Zhou Z, Alégot H, Irvine KD (2019). Oriented cell divisions are not required for *Drosophila* wing shape. Curr Biol.

