# Supplementary material for: Epithelial tissue folding pattern in confined geometry
Source: Biomech Model Mechanobiol. 2019 Nov 14;19(3):815–22. doi: 10.1007/s10237-019-01249-8 (PMC7203093; doi:10.1007/s10237-019-01249-8)
Supplement: Supplementary file 1 — Supplementary material 1 (pdf 151 KB) [file 10237_2019_1249_MOESM1_ESM.pdf]

Supplementary Material

Movie 1(ESM_1.mp4): There is no notable tissue deformation even after equilibration for 0.5 cell cycle from the initial condition.

Movie 2(ESM_2.mp4): Epithelial folding simulated under the condition of restraint of out-of-plane deformation ($k^{\mathrm{r}} = 1 \times 10^{-3}$). Division axis is the longest axis of each cell.

Movie 3(ESM_3.mp4): Epithelial folding simulated under the condition of no restraint of out-of-plane deformation ($k^{\mathrm{r}} = 0$). Division axis is the longest axis of each cell.

Movie 4(ESM_4.mp4): Epithelial folding simulated under the condition of restraint of out-of-plane deformation ($k^{\mathrm{r}} = 1 \times 10^{-3}$). Division axis is the x-axis.

Movie 5(ESM_5.mp4): Epithelial folding simulated under the condition of restraint of out-of-plane deformation ($k^{\mathrm{r}} = 1 \times 10^{-3}$). Division axis is the radial axis.
